# A Randomized Trial Assessing the Safety, Pharmacokinetics, and Efficacy During Morning *Off* of AZ‐009

**DOI:** 10.1002/mds.28926

**Published:** 2022-01-20

**Authors:** Eva Thijssen, Jonas den Heijer, David Puibert, Laurence Moss, Mingzu Lei, David Hasegawa, Kyo Keum, Ken Mochel, Mohammed Ezzeldin Sharaf, Tom Alfredson, Wenxiang Zeng, Emilie van Brummelen, Tatjana Naranda, Geert Jan Groeneveld

**Affiliations:** ^1^ Centre for Human Drug Research (CHDR) Leiden the Netherlands; ^2^ Leiden University Medical Centre (LUMC) Leiden the Netherlands; ^3^ Ferrer Barcelona Spain; ^4^ Alexza Pharmaceuticals Mountain View California USA

**Keywords:** Parkinson's disease, AZ‐009, apomorphine, inhalation, randomized clinical trial, *off*

## Abstract

**Background:**

Inhalation of apomorphine could be a faster‐acting and more user‐friendly alternative to subcutaneous injection for treating *off* periods in Parkinson's disease (PD).

**Objective:**

The aim of this study was to compare the safety and pharmacokinetics of inhaled apomorphine (AZ‐009) with subcutaneous apomorphine (APO‐go PEN) in healthy volunteers (HVs) and to examine the safety, pharmacokinetics, and efficacy of AZ‐009 in patients with PD.

**Methods:**

In part A of this study, eight HVs received 1 mg AZ‐009 and 2 mg subcutaneous apomorphine in a randomized crossover manner. In the subsequent single ascending dose parts in HVs (part B, n = 16) and patients with PD (part C, n = 25), participants were randomized to placebo or AZ‐009 up to 4 mg. In patients, after medication withdrawal, Movement Disorder Society‐Unified Parkinson's Disease Rating Scale part III and *on/off* states were assessed predose and postdose.

**Results:**

AZ‐009 was rapidly absorbed with peak plasma concentrations at 2 minutes, as compared to 30 minutes for subcutaneous apomorphine. Adverse events for AZ‐009 were comparable to subcutaneous apomorphine, except for mild and transient throat irritation. Adverse events limited AZ‐009 dose escalation in HVs to 3 mg. Patients tolerated up to 4 mg. In patients with PD, 2, 3, and 4 mg AZ‐009 reduced mean Movement Disorder Society‐Unified Parkinson's Disease Rating Scale part III score (standard deviation) by 10.7 (13.6), 12.8 (7.9), and 10.3 (3.7) points, respectively, compared to 4.8 (4.9) after placebo at 10 minutes postdose. The percentage of patients achieving full *on* within 45 minutes postdose increased dose dependently: 0% (placebo), 17% (2 mg), 50% (3 mg), and 83% (4 mg).

**Conclusions:**

AZ‐009 appears to be a rapid‐acting and reasonably well‐tolerated formulation for treating *off* periods. © 2022 The Authors. *Movement Disorders* published by Wiley Periodicals LLC on behalf of International Parkinson and Movement Disorder Society

Parkinson's disease (PD) is a progressive neurodegenerative disorder that affects movement, cognition, emotion, and autonomic activity. Patients with PD are usually treated with dopaminergic drugs, such as levodopa (l‐dopa) and/or a direct‐acting dopamine agonist. Initial therapy is selected based on a number of criteria, including patient age, comorbid conditions, disease severity, and degree of functional disability.^1^, ^2^, ^3^ However, most patients eventually require l‐dopa therapy, and a large proportion of patients experience motor complications within a few years of starting its use.^4^, ^5^, ^6^ Complications consist of predictable end‐of‐dose *off* episodes (“wearing *off*”), prolonged latency to *on*, inability to turn *on*, sudden *on/off* fluctuations, and/or dyskinesia. These fluctuations in therapeutic effects can be predictable or unpredictable and do not only involve fluctuations in motor symptoms but also in nonmotor symptoms, such as anxiety/panic attacks, mood changes, slow thinking, and pain.^7^

A number of strategies have been investigated to increase *on* time while reducing disabling *off* time, eg, dosing more often with a lower l‐dopa dose, adding dopamine agonists, giving catechol‐*O*‐methyltransferase or monoamine oxidase B inhibitors, administering controlled‐ or sustained‐release drug formulations, or following a protein redistribution diet.^2^, ^8^, ^9^ However, despite optimal oral therapy, patients often continue to experience *off* periods that severely compromise quality of life and daily activities.^10^ Subcutaneous apomorphine provides rapid and effective relief from such *off* periods and has been indicated for use in advanced PD for approximately two decades. Often reported side effects include injection‐site reactions, hallucinations, sedation, somnolence, dizziness, yawning, and nausea and vomiting. In addition, there is an increased risk of orthostatic hypotension in the elderly population, especially during initiation of therapy.^11^ To diminish the risk of nausea, vomiting, and (orthostatic) hypotension, patients are usually pretreated with domperidone or another antiemetic for at least 2 days before initiation of apomorphine.^11^, ^12^, ^13^ Although the subcutaneous formulation of apomorphine is efficacious, it has disadvantages, such as difficulty self‐administering a subcutaneous injection while *off* and a high incidence of injection‐site reactions.^14^ A more user‐friendly formulation would allow for a broader use of apomorphine. This unmet medical need is recognized by the medical community, and research has been focused on finding more suitable formulations.^14^, ^15^ Recently, sublingual apomorphine has been approved by the U.S. Food and Drug Administration, providing a more user‐friendly formulation, albeit still requiring a film strip under the tongue for up to 3 minutes.^16^ It is expected that apomorphine inhalation will not only be more user‐friendly but also result in an even faster action.

AZ‐009, also called Staccato apomorphine, is a single‐use, disposable, breath‐actuated drug‐device combination product for oral inhalation. It has been developed to deliver apomorphine hydrochloride as a thermally generated, condensation aerosol to the deep lung for rapid systemic exposure. We performed a three‐part phase 1 trial to evaluate the pharmacokinetics (PK) of AZ‐009 and compare it with a registered subcutaneous apomorphine injection (part A) and to study the safety and PK of single ascending doses of AZ‐009 in healthy volunteers (HVs) (part B) and patients with PD (part C). The last study part also evaluated the efficacy of AZ‐009 during an induced morning *off* state.

## Subjects and Methods

The study was conducted in accordance with European Medicines Agency guidelines for Good Clinical Practice and registered in ClinicalTrials.gov (NCT03822364). The protocol was approved by the Independent Ethics Committee of Foundation Beoordeling Ethiek Biomedisch Onderzoek. Before any study‐related activity, all participants provided written informed consent. The study was conducted at the Centre for Human Drug Research between October 2018 and May 2019.

### Study Design

This study was divided into three parts: parts A, B, and C. Refer to Supporting Information Fig. S1 for a schematic overview of the study designs. The randomization code was generated separately for each part using SAS version 9.4 by a study‐independent Centre for Human Drug Research statistician. No formal sample size calculations were performed. Part A of the study was a randomized, open‐label crossover study assessing single doses of AZ‐009 (1 mg) and subcutaneous apomorphine (2 mg) in eight HVs. The washout between the two study periods was at least 3 days (apomorphine half‐life is approximately 30–50 minutes^17^, ^18^). Safety data were examined during a dose‐level evaluation meeting before proceeding to study part B. Part B was a randomized, double‐blind, placebo‐controlled, single ascending dose study of AZ‐009 with planned doses of 2, 3, and 4 mg in HVs. The 4‐mg cohort was canceled because of incidence of adverse events (AEs) in the 3‐mg cohort. Each cohort was composed of eight HVs of which six were randomized to receive active treatment and two to receive placebo. Before advancing to the next cohort, safety data were evaluated. Part C had the same study design as part B but was performed in patients with PD after overnight anti‐Parkinson's medication withdrawal. Patients were dosed the next morning only when they were in an *off* state as assessed by a physician.

The study consisted of a screening visit; at‐home pretreatment with an antiemetic (domperidone) three times daily; a single stay of 7, 3, or 2 days (parts A, B, and C, respectively) at the clinical research unit; and a follow‐up telephone call. In part A, participants received 10 mg domperidone three times daily from 3 days before dosing until after last dose. In part B, domperidone dose was increased to 20 mg on the evening and morning before dosing. At other time points, domperidone intake remained 10 mg as in part A. In part C, participants received 20 mg domperidone three times daily from 2 days before dosing until after dosing.

### Participants

In study parts A and B, healthy nonsmoking men and women aged 18–60 years with a body mass index of 18–32 kg/m^2^ were eligible to participate. In study part C, nonsmoking patients with PD with recognizable *off* periods aged 30–85 years with Hoehn & Yahr stage I–IV were eligible for participation. Patients were excluded if their systolic blood pressure (BP) was below 100 mm Hg at screening or baseline, they had symptomatic clinically relevant and medically uncontrolled orthostatic hypotension, or a history of long QT syndrome and/or a QTcF of >470 (male) or >480 ms (female).

### Investigational Drugs

AZ‐009 was available in two dose strengths (1 and 2 mg apomorphine hydrochloride). A dose of 3 mg was delivered by three consecutive oral inhalations of 1 mg and a dose of 4 mg by two consecutive inhalations of 2 mg. Matching Staccato placebo (including number of devices inhaled) was identical to AZ‐009 but without a coated apomorphine film. AZ‐009 and matching placebo were manufactured by Alexza Pharmaceuticals Inc. Participants were instructed to inhale through the mouthpiece with a steady deep breath and to hold their breath for as long as possible, up to 10 seconds.

Inhalation through the product initiates the controlled rapid heating of a thin film of excipient‐free apomorphine to form a thermally generated drug vapor. The vapor condenses into aerosol particles with a particle size distribution appropriate for efficient delivery to the deep lung, ie, with a mass median aerodynamic diameter in the range of 0.5 to 3.5 μm.

In study part A, apomorphine was also administered subcutaneously with the APO‐go PEN. APO‐go was provided as the commercially available product with the appropriate country‐specific labeling by the Leiden University Medical Centre pharmacy. A volume of 0.2 mL (2 mg) was injected in the thigh.

### Safety

For all study parts, a medical screening was performed to assess eligibility based on medical history, concomitant medications, electrocardiogram, vital signs, routine hematology, chemistry and urinalysis, and physical examination. Electrolytes and QTcF were assessed at screening (before domperidone initiation) and again at baseline (after domperidone initiation and before apomorphine administration). During the study, safety was evaluated by monitoring of AEs (classified by Medical Dictionary for Regulatory Activities [MedDRA] version 20.1), vital signs, electrocardiograms, physical examination, and clinical laboratory tests. Orthostatic hypotension was defined as a systolic BP decline of ≥20 mm Hg or a diastolic BP decline of ≥10 mm Hg on standing. Postural dizziness was defined as dizziness on standing that was not accompanied by a decline in BP (at the scheduled measurement time) as defined for orthostatic hypotension.

### Pharmacokinetics

Blood samples for PK analysis were obtained predose; at 1, 2, 5, 10, 20, 30, and 45 minutes postdose; and 1, 2, 4, 8, and 24 hours postdose in parts A and B. In part C, samples were obtained predose; at 2, 5, 15, 30, and 45 minutes postdose; and at 1, 1.5, 4, and 5 hours postdose. A lower sampling frequency and shorter sampling duration were chosen in part C to allow time for efficacy measurements and to reduce patient burden. Plasma samples were analyzed for apomorphine using a validated liquid chromatography–tandem mass spectrometry method.

Plasma concentrations of apomorphine were analyzed using noncompartmental analysis in Phoenix WinNonlin version 8.1. PK parameters that were calculated include maximum plasma concentration (*C*_max_), time to *C*_max_ (*T*_max_), apparent terminal elimination half‐life (*t*_1/2_), and area under the plasma concentration time curve from zero to infinity (AUC_0‐inf_).

For part A, the comparison of the dose‐normalized log‐transformed PK parameters *C*_max_ and AUC_0‐inf_ for apomorphine across treatments (1 mg AZ‐009 inhalation vs. 2 mg subcutaneous apomorphine) was performed using an analysis of variance model and the two one‐sided *t* tests procedure. The analysis of variance model included factors for sequence, subject within sequence, treatment, and period. Point estimates and 90% confidence intervals for the geometric mean ratios (AZ‐009/subcutaneous apomorphine) were calculated for PK parameters by back transformation to the original scale.

For parts A to C combined, *C*_max_ and AUC_0‐inf_ for apomorphine were compared across dose levels (1–4 mg) to assess dose proportionality. Statistical analyses were conducted using a power model with mixed effects.^19^

### Efficacy

Motor function was assessed using part III of the licensed Movement Disorder Society‐Unified Parkinson's Disease Rating Scale (MDS‐UPDRS). Physicians administering the scale were trained and certified in its use. To the degree feasible, the same physician evaluated a patient at day −1 (day before dosing), day 1 predose, and 10, 30, and 60 minutes postdose. Mean change from baseline MDS‐UPDRS part III total score was calculated and presented graphically.

The disease state of a patient was assessed by a physician predose and at 2, 10, 20, and 45 minutes postdose. Possible categories were *on* with disabling dyskinesia, *on* with nondisabling dyskinesia, *on* with no dyskinesia and normal motor function, partial *on* and *off*. The first three categories were combined, classified as full *on*, and presented graphically as percentage of patients turning full *on*.

## Results

### Demographics

See Supporting Information Figs. [Link], [Link] for CONSORT flow diagrams providing an overview of number of participants screened, randomized, completed, and analyzed per study part. Table 1 outlines the demographics and disposition of all participants enrolled in the study. Eight HVs completed the comparative PK study part (part A), and two cohorts of eight HVs (six AZ‐009, two placebo) completed the single ascending‐dose study part (part B). Demographics of HVs in part A and B were comparable, only the median age was higher in part A compared with part B (40 and 26 years, respectively). In part C of the study, a total of 25 patients with PD were included, divided over three cohorts receiving 2, 3, or 4 mg AZ‐009 or placebo in a 6:2 ratio. The 2 mg AZ‐009 group contained one additional patient because of a replacement in cohort 1 (see Supporting Information Fig. S4). The age of patients with PD was higher than that of HVs. All groups contained men and women, except for the placebo group, which was composed of men only.

**TABLE 1:** Demographics of participants in study parts A to C

Demographic variables for healthy volunteers	Part A	Part B			
	All participants (n = 8)	All participants (n = 16)	2 mg AZ‐009 (n = 6)	3 mg AZ‐009 (n = 6)	Placebo (n = 4)
Age (y), median (range)	40 (19–58)	26 (19–60)	29 (21–39)	24 (21–60)	40 (19–58)
BMI (kg/m^2^), median (range)	25 (20–31)	24 (19–30)	24 (19–28)	24 (21–27)	26 (24–30)
Sex, female/male, n/n (%/%)	5/3 (62.5/37.5)	12/4 (75.0/25.0)	5/1 (83.3/16.7)	5/1 (83.3/16.7)	2/2 (50.0/50.0)
Race, n (%)					
Asian	0 (0)	2 (12.5)	1 (16.7)	1 (16.7)	0 (0)
Mixed	2 (25.0)	2 (12.5)	2 (33.3)	0 (0)	0 (0)
White	6 (75.0)	12 (75.0)	3 (50.0)	5 (83.3)	4 (100.0)

Demographic variables for patients with PD	Part C				
	All participants (n = 24)^a^ (n = 25)^b^	2 mg AZ‐009 (n = 6)^a^ (n = 7)^b^	3 mg AZ‐009 (n = 6)	4 mg AZ‐009 (n = 6)	Placebo (n = 6)
Age (y), median (range)	62 (44–83)	63 (58–75)	55 (53–67)	67 (56–71)	58 (44–83)
BMI (kg/m^2^), median (range)	25 (20–31)^a^ 25 (20–32)^b^	27 (20–30)^a^ 27 (20–32)^b^	26 (22–29)	24 (22–27)	24 (22–31)
Sex, female/male, n (%/%)	7/17 (29.2/70.8)^a^ 7/18 (28.0/72.0)^b^	3/3 (50.0/50.0)^a^ 3/4 (42.9/57.1)^b^	1/5 (16.7/83.3)	3/3 (50.0/50.0)	0/6 (0/100.0)
Race, n (%)					
Other^c^	1 (4.2^a^, 4.0^b^)	0 (0)	1 (16.7)	0 (0)	0 (0)
White	23 (95.8)^a^ 24 (96.0)^b^	6 (100.0)^a^ 7 (100.0)^b^	5 (83.3)	6 (100.0)	6 (100.0)
MMSE, median (range)	29 (25–30)	30 (27–30)	29 (27–30)	30 (25–30)	29 (26–30)
Hoehn & Yahr stage at day −1 (when using regular medication), n (%)					
Stage 1	1 (4.2)	0 (0)	0 (0)	0 (0)	1 (16.7)
Stage 2	15 (62.5)	5 (83.3)	3 (50.0)	4 (66.7)	3 (50.0)
Stage 3	6 (25.0)	1 (16.7)	2 (33.3)	2 (33.3)	1 (16.7)
Stage 4	2 (8.3)	0 (0)	1 (16.7)	0 (0)	1 (16.7)
MDS‐UPDRS Part III total score at day −1 (when using regular medication)					
Median (range)	33 (13–76)	30 (15–38)	36 (19–73)	30 (22–50)	32 (13–76)
Concomitant PD medication, n (%)					
Levodopa‐containing agents	23 (95.8)	6 (100.0)	6 (100.0)	6 (100.0)	5 (83.3)
Dopamine agonists	16 (66.7)	5 (83.3)	5 (83.3)	2 (33.3)	4 (66.7)
COMT inhibitors	7 (29.2)	3 (50.0)	2 (33.3)	2 (33.3)	0 (0)
MAO‐B inhibitors	3 (12.5)	2 (33.3)	1 (16.7)	0 (0)	0 (0)
Amantadine	4 (16.7)	2 (33.3)	1 (16.7)	1 (16.7)	0 (0)

In part C, when the pharmacodynamics population differed from the pharmacokinetics/safety population in age, BMI, sex, and/or race, information is provided for both; remaining variables are presented for the pharmacodynamics population only.

^a^ Information given for pharmacodynamics analysis population.

^b^ Information given for pharmacokinetics and safety analysis population.

^c^ North African.

BMI, body mass index; MMSE, Mini Mental State Examination; MDS‐UPDRS, Movement Disorder Society–Unified Parkinson's Disease Rating Scale; PD, Parkinson's disease; COMT, catechol‐*O*‐methyltransferase; MAO‐B, monoamine oxidase B.

### Pharmacokinetics

#### Part A: Comparative PK in HVs

**FIG 1:**
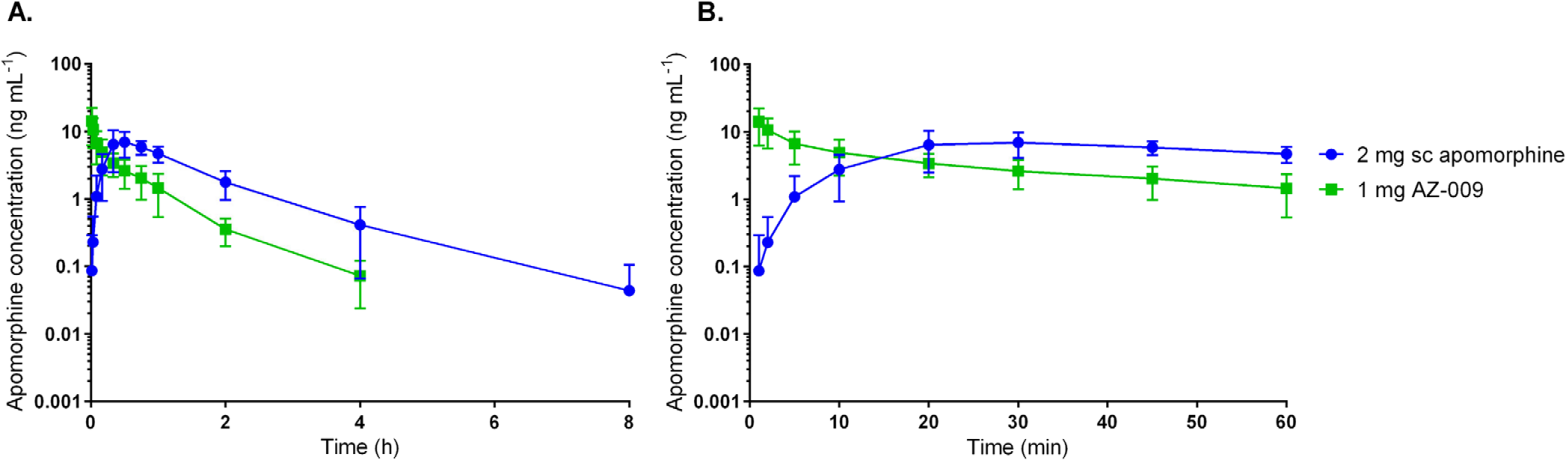
Mean (standard deviation) apomorphine concentration time profiles after single‐dose administrations of 1 mg AZ‐009 and 2 mg subcutaneous (sc) apomorphine on semilogarithmic scale to healthy volunteers up to 8 hours (**A**) and 1 hour (**B**) postdose. [Color figure can be viewed at wileyonlinelibrary.com]

Apomorphine was rapidly absorbed into the systemic circulation after administration of AZ‐009 and subcutaneous apomorphine in HVs (Fig. 1). Descriptive statistics of the PK parameters are summarized in Supporting Information Table S1. AZ‐009 inhalation resulted in *C*_max_ 1 to 2 minutes after dosing and showed a biexponential elimination phase. In contrast, apomorphine concentrations after subcutaneous apomorphine injection increased over time with a median *T*_max_ of 30 minutes. When normalized for dose, the *C*_max_ and AUC_0‐inf_ geometric mean ratios (90% confidence interval) of AZ‐009/subcutaneous apomorphine were 2.9 (1.6–5.4) and 0.8 (0.5–1.2), respectively. Mean apomorphine *t*_1/2_ ± standard deviation (SD) of AZ‐009 was shorter (39 ± 7 minutes) than that of subcutaneous apomorphine (55 ± 22 minutes). Intersubject variability (CV%) in apomorphine *C*_max_ and AUC_0‐inf_ was higher for AZ‐009 (53.7% and 47.2%) than for subcutaneous apomorphine (36.4% and 22.7%).

#### Parts B and C: Single Ascending Doses in HVs and Patients with PD

**FIG 2:**
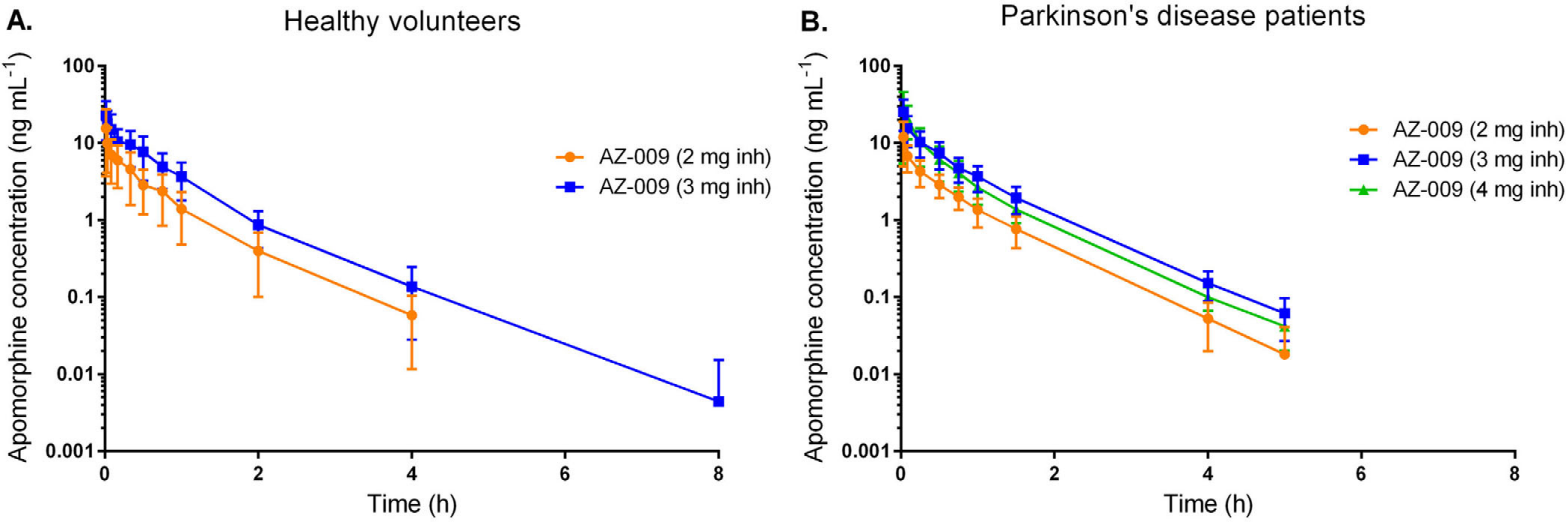
Mean (standard deviation) apomorphine concentration time profiles after single‐dose administrations of 2 or 3 mg AZ‐009 to healthy volunteers (part B) (**A**) and 2, 3, or 4 mg AZ‐009 to patients with PD (part C) (**B**) on a semilogarithmic scale. [Color figure can be viewed at wileyonlinelibrary.com]

AZ‐009 was rapidly systemically absorbed in HVs (Fig. 2A), as well as in patients with PD (Fig. 2B). Median *T*_max_ in HVs was similar as in part A, ie, 1 minute. The first PK sample in patient with PD was taken at 2 minutes postdose. Median *T*_max_ in patients with PD was 2 or 3 minutes depending on the dose group (Supporting Information Table S2). *C*_max_ and AUC_0‐inf_ after 2 and 3 mg AZ‐009 were similar for HVs and patients with PD. *t*_1/2_ in both HVs and patients with PD was similar as was reported for 1 mg AZ‐009 in part A. In patients with PD, AUC_0‐inf_ increased from 2 to 3 mg, but not from 3 to 4 mg, ie, mean (SD) AUC_0‐inf_ was 5.1 (1.5), 12.6 (4.5), and 11.3 (5.1) h/ng/mL for 2, 3, and 4 mg AZ‐009, respectively.

Dose proportionality was assessed on the combined data of parts A to C. The estimated exponent (90% confidence interval) was 0.57 (0.15–1.00) for the *C*_max_ and 0.77 (0.41–1.13) for the AUC_0‐inf_.

### Safety and Tolerability

The incidence of moderate AEs was 62.5% after AZ‐009 and 100% after subcutaneous apomorphine treatment (Table 2). The most frequently reported treatment‐emergent adverse events (TEAEs) were nausea and presyncope (despite pretreatment with 10 mg domperidone three times daily) and somnolence and headache. Participants who received subcutaneous apomorphine reported the first AEs around 20 minutes postdose, whereas for AZ‐009 this was after 2 to 3 minutes (data not shown).

**TABLE 2:** Summary of the number of TEAEs and the number and percentage of participants (n [%]) with any, mild, moderate, and severe TEAEs and with a specific TEAE as indicated per treatment group and study part

	Part A: crossover study in HVs, n (%)		Part B: SAD study in HVs, n (%)			Part C: SAD study in patients with PD, n (%)			
	2 mg sc apo (n = 8), n (%)	1 mg AZ‐009 (n = 8), n (%)	2 mg AZ‐009 (n = 6), n (%)	3 mg AZ‐009 (n = 6), n (%)	Placebo (n = 4), n (%)	2 mg AZ‐009 (n = 7), n (%)	3 mg AZ‐009 (n = 6), n (%)	4 mg AZ‐009 (n = 6), n (%)	Placebo (n = 6), n (%)
No. of TEAEs^a^	43	35	13	43	0	23	24	24	10
Any TEAEs	8 (100.0)	7 (87.5)	6 (100.0)	6 (100.0)	‐	6 (85.7)	5 (83.3)	6 (100.0)	5 (83.3)
Mild TEAEs	7 (87.5)	7 (87.5)	5 (83.3)	6 (100.0)	‐	6 (85.7)	5 (83.3)	6 (100.0)	4 (66.7)
Moderate TEAEs	8 (100.0)	5 (62.5)	3 (50.0)	5 (83.3)	‐	3 (42.9)	1 (16.7)	2 (33.3)	1 (16.7)
Severe TEAEs	‐	‐	‐	‐	‐	‐	1 (16.7)	‐	‐
Most common TEAEs^b^									
Lacrimation increased	‐	‐	1 (16.7)	2 (33.3)	‐	1 (14.3)	‐	1 (16.7)	‐
Nausea	6 (75.0)	5 (62.5)	1 (16.7)	5 (83.3)	‐	1 (14.3)	1 (16.7)	2 (33.3)	1 (16.7)
Vomiting	4 (50.0)	1 (12.5)	‐	1 (16.7)	‐	‐	‐	‐	‐
Throat irritation	‐	‐	1 (16.7)	2 (33.3)	‐	2 (28.6)	2 (33.3)	5 (83.3)	‐
Fatigue	2 (25.0)	1 (12.5)	1 (16.7)	‐	‐	1 (14.3)	2 (33.3)	‐	1 (16.7)
Feeling hot	3 (37.5)	2 (25.0)	‐	1 (16.7)	‐	1 (14.3)	2 (33.3)	‐	‐
Sluggishness	1 (12.5)	1 (12.5)	‐	‐	‐	‐	‐	‐	‐
Dizziness	3 (37.5)	2 (25.0)	‐	2 (33.3)	‐	1 (14.3)	1 (16.7)	1 (16.7)	‐
Headache	3 (37.5)	4 (50.0)	‐	‐	‐	‐	1 (16.7)	‐	‐
Orthostatic hypotension									
Asymptomatic	1 (12.5)	‐	‐	‐	‐	2 (28.6)	2 (33.3)	2 (33.3)	4 (66.7)
Symptomatic	2 (25.0)	‐	‐	4 (66.7)	‐	1 (14.3)	‐	‐	‐
Dizziness postural^c^	2 (25.0)	3 (37.5)	‐	1 (16.7)	‐	1 (14.3)	1 (16.7)	1 (16.7)	‐
Increased PD symptoms	‐	‐	‐	‐	‐	2 (28.6)	‐	2 (33.3)	2 (33.3)
Presyncope	5 (62.5)	3 (37.5)	‐	3 (50.0)	‐	2 (28.6)	‐	1 (16.7)	‐
Somnolence	5 (62.5)	3 (37.5)	3 (50.0)	5 (83.3)	‐	2 (28.6)	1 (16.7)	1 (16.7)	‐
Syncope	1 (12.5)	1 (12.5)	‐	‐		‐	1 (16.7)	‐	‐
Time perception altered	1 (12.5)	2 (25.0)	‐	2 (33.3)	‐	‐	1 (16.7)	‐	‐
Yawning	‐	1 (12.5)	2 (33.3)	4 (66.7)	‐	2 (28.6)	2 (33.3)	1 (16.7)	‐

^a^ Not expressed as n (%). This parameter describes the total number of TEAEs reported, and hence is unitless.

^b^ TEAEs reported by at least two (part A/B) or three (part C) of the apomorphine‐treated participants.

^c^ Dizziness on standing but no significant blood pressure decline measured at scheduled standing blood pressure measurement.

TEAE, treatment‐emergent adverse event; sc apo, subcutaneous apomorphine; HV, healthy volunteer; SAD, single ascending dose; PD, Parkinson's disease.

In part B, the domperidone dose was increased to 20 mg on the evening and morning before dosing in HVs. At other time points, domperidone intake remained 10 mg. A dose of 2 mg AZ‐009 combined with this higher domperidone dose was better tolerated than 1 mg AZ‐009 combined with a lower dose of domperidone (Table 2). The most frequently reported TEAEs were somnolence and yawning. The number of TEAEs, and in particular the frequency of moderate TEAEs, increased from 2 to 3 mg AZ‐009. Nausea, orthostatic hypotension, somnolence, and yawning were reported most often in the 3‐mg group. Standing BPs as low as 70/34 mm Hg were measured, and five of six participants in the 3‐mg group needed to lie down until symptoms subsided. Due to the dose‐dependent increase in incidence of TEAEs, it was decided not to escalate to 4 mg in HVs and to increase the domperidone dose to 20 mg three times daily from 2 days before dosing in part C of the study in patients with PD.^12^, ^13^

AZ‐009 was relatively well tolerated by patients with PD at 2, 3, and 4 mg with mostly mild TEAEs (Table 2). The most frequently reported TEAEs in the AZ‐009–treated groups were throat irritation, orthostatic hypotension, and yawning. Orthostatic hypotension was mostly asymptomatic and was also reported in the placebo group. Some patients reported an increase in their PD symptoms in the days after the overnight Parkinson's medication withdrawal and dosing with placebo or AZ‐009. No increase in incidence and severity of TEAEs was observed with an increase in dose. Most TEAEs resolved without treatment, except for one case of severe hypotension in the 3‐mg group that was treated with ephedrine, and two cases where the number of PD medication doses was increased for several days after study participation because of increased PD symptoms.

No consistent or clinically relevant QTcF prolongation or clinical laboratory changes were reported in any of the participants.

### Efficacy

**FIG 3:**
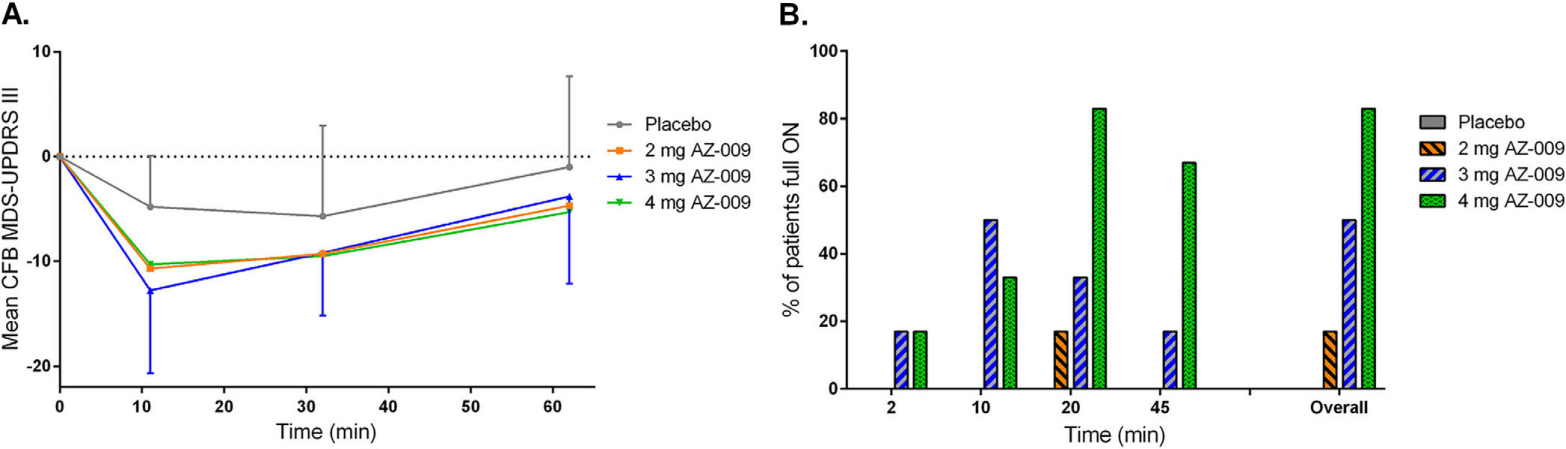
Mean change from baseline (CFB) Movement Disorder Society‐Unified Parkinson's Disease Rating Scale (MDS‐UPDRS) part III total score with standard deviation (**A**) and percentage (%) of patients achieving a full *on* response (**B**) after the indicated treatment in patients with PD during an induced *off* state. [Color figure can be viewed at wileyonlinelibrary.com]

Patients with PD in part B were dosed during an *off* state after overnight medication withdrawal. All three AZ‐009–treated dose groups showed a reduction from baseline in mean MDS‐UPDRS part III total score at the first assessment 10 minutes postdose (Fig. 3A). The mean MDS‐UPDRS part III change from baseline with SD at this time point was −10.7 (13.6) for the 2‐mg group, −12.8 (7.9) for the 3‐mg group, −10.3 (3.7) for the 4‐mg group, and −4.8 (4.9) for the placebo group. The effect observed in the AZ‐009–treated groups started to decrease at 30 minutes postdose and further decreased at 1 hour postdose to less than half of the maximum effect observed at 10 minutes postdose. In contrast, the placebo group no longer showed a reduction compared with baseline at 1 hour postdose.

All patients were assessed by a physician as being in an *off* state before dosing (Fig. 3B). None of the placebo‐treated patients achieved a full *on* response at any of the time points. In contrast, the first patients converted to a full *on* as early as 2 minutes after AZ‐009 dosing. The highest percentage of patients in an *on* state occurred 10 minutes postdose for the 3 mg AZ‐009 group and 20 minutes post‐dose for the 2 and 4 mg AZ‐009 groups. The percentage of patients achieving a full *on* at any time point within 45 minutes postdose increased with dose from 17% (2 mg) to 50% (3 mg) to 83% (4 mg). No patients presented with disabling dyskinesias.

## Discussion

Subcutaneous apomorphine injections have long been used by patients with PD for the treatment of sudden or early‐morning *off* periods. Even though subcutaneous apomorphine is efficacious, it can be painful and/or difficult to self‐administer and often results in injection‐site reactions.^11^ Moreover, maximal motor improvements have been shown to occur only after about 20 to 40 minutes after subcutaneous apomorphine.^20^, ^21^, ^22^ This formulation of inhalable apomorphine, AZ‐009, could provide an easier and faster‐acting formulation for the treatment of *off* periods. This three‐part study was designed to evaluate the PK of AZ‐009 and compare it with the subcutaneous injection and to examine the safety and PK of ascending doses of AZ‐009 in HVs and patients with PD. The last study part also aimed to evaluate the efficacy of AZ‐009 in patients with PD during an induced morning *off* state.

AZ‐009 led to rapid systemic exposure with a median *T*_max_ of 2 minutes based on the combined data of HVs and patients with PD. In contrast, the subcutaneous apomorphine injection resulted in a *T*_max_ of 30 minutes. The PK profile of AZ‐009 makes it especially suitable for fast onset of action, which is preferential in the treatment of sudden *off* periods. Dosing with 1 mg AZ‐009 resulted in a mean (SD) *C*_max_ of 14.3 (7.7) ng/mL and 2 mg subcutaneous apomorphine in 8.6 (3.1) ng/mL. A difference in total exposure (AUC_0‐inf_) between inhalable and subcutaneous apomorphine could not be confirmed because of the relatively high variability and small sample size. Similarly, no definitive conclusions could be drawn on dose proportionality. Future larger trials will need to be conducted to gain more information on this.

Despite comparable PK, AZ‐009 resulted in a less favorable safety profile in HVs than in patients with PD. This was not unexpected because patients with PD are likely to have developed tolerance because of daily dopaminergic medication use. Also, patients with PD were administered a higher domperidone dose compared with HVs. The most frequently reported AEs in patients with PD were throat irritation, orthostatic hypotension, and yawning. Throat irritation occurred immediately after dosing and usually resolved within minutes. Orthostatic hypotension was mostly asymptomatic and was observed in the placebo group as well. This can likely be partly explained by autonomic dysregulation in PD.

One patient with PD receiving 3 mg AZ‐009 presented with severe hypotension shortly after dosing that was treated with ephedrine. Hypotension is a known side effect of apomorphine,^12^, ^23^ and moderate hypotension was also reported by one HV receiving 2 mg subcutaneous apomorphine in study part A. All participants who presented with reduced BP spontaneously recovered after lying down or lying in Trendelenburg position. However, in the context of patient comfort, ephedrine was more readily administered during the patient part of the study. Moreover, AZ‐009 gives higher peak apomorphine concentrations than subcutaneous apomorphine, and this patient was immediately given 3 mg AZ‐009. In clinical practice, subcutaneous apomorphine is initiated under medical supervision at 2 mg and titrated up to a dose that is both tolerable and effective. The same should be done with AZ‐009 when used in clinical practice. For some patients, AZ‐009 might not be tolerable at effective doses, as is now also the case for some patients receiving subcutaneous injections.

During this trial, a prototype of the inhalation device was used. Of 25 patients with PD, 23 (92.0%) indicated they liked how the drug was delivered. Whether they also found the device easy to use could not be adequately evaluated because of the prototype being used. Future trials should therefore focus on ease of use of the commercial device in patients with PD.

Treatment with 2, 3, and 4 mg AZ‐009 showed promise in controlling morning *off* periods in patients with PD after overnight medication withdrawal. At 10 minutes postdose, all three AZ‐009 dose groups showed a clear reduction (10.3–12.8 points) from baseline in mean MDS‐UPDRS part III score that was greater than for placebo (4.8 points). These reductions were larger than 3.25 points, which has been described as the minimal but clinically relevant improvement.^24^ Moreover, the difference in MDS‐UPDRS part III response between placebo and apomorphine was comparable with that reported in another apomorphine inhalation study (8.4 points [95% confidence interval: 1.2–15.5]).^25^ MDS‐UPDRS part III improvement did not seem to correlate with AZ‐009 dose. This is likely the result of interpatient variability in exposure and MDS‐UPDRS part III response. From literature, it was already known that the minimally effective apomorphine concentration differs widely between patients,^26^ and that the degree of response is (partly) dependent on disease severity.^27^ The fast onset of action and relatively short duration of action would make this formulation ideal for patients suffering from sudden and unpredictable *off* periods or from delayed *on*. Findings on the MDS‐UPDRS part III were supported by the physician's *on*/*off* state assessment. Whereas none of the placebo patients achieved a full *on* response, the AZ‐009–treated patients dose‐dependently converted from *off* to full *on*. For future studies, assessing *on*/*off* states after 45 minutes is advised to determine duration of clinical effect. Because patients were randomized to their AZ‐009 dose, it is likely that they did not reach their maximum possible improvement. In clinical practice, the dose of apomorphine is titrated to reach a dose with optimal efficacy and minimal side effects. Whereas this study demonstrates a beneficial effect of AZ‐009 over placebo, future studies should further investigate the efficacy of AZ‐009 at the patient's optimal dose.

Taken together, AZ‐009 is reasonably well tolerated by patients with PD pretreated with domperidone. AZ‐009 is rapidly absorbed into the systemic circulation and can provide rapid relief from early‐morning *off* periods.

## Author Roles

1. Research project: A. Conception, B. Organization, C. Execution;

2. Statistical Analysis: A. Design, B. Execution, C. Review and Critique;

3. Manuscript: A. Writing of the first draft, B. Review and Critique.

E.T.: 1A, 1B, 1C, 2A, 2C, 3A

J.d.H.: 1A, 1B, 1C, 2A, 2C, 3B

D.P.: 1A, 1B, 2A, 2C, 3B

L.M.: 1C, 3B

M.L.: 3B

D.H.: 3B

K.K.: 3B

K.M.: 3B

M.E.S.: 3B

T.A.: 3B

W.Z.: 3B

E.v.B.: 1A, 1B, 2A, 2C, 3B

T.N.: 1A, 1B, 2A, 2C, 3B

G.J.G.: 1A, 1B, 1C, 2A, 2C, 3B

## Financial Disclosures of All Authors (for the Preceding 12 Months)

Eva Thijssen: Employee, Centre for Human Drug Research (CHDR) and Leiden University Medical Centre (LUMC), Leiden, the Netherlands. Jonas den Heijer: Employee, CHDR and LUMC, Leiden, the Netherlands. David Puibert: Employee, Ferrer, Barcelona, Spain. Laurence Moss: Employee, CHDR and LUMC, Leiden, the Netherlands. Mingzu Lei: Employee, Alexza Pharmaceuticals, Mountain View, CA, USA. David Hasegawa: Employee, Alexza Pharmaceuticals, Mountain View, CA, USA. Kyo Keum: Alexza Pharmaceuticals, Mountain View, CA, USA. Ken Mochel: Employee, Alexza Pharmaceuticals, Mountain View, CA, USA. Mohammed Ezzeldin Sharaf: Employee, Ferrer, Barcelona, Spain. Tom Alfredson: Employee, Alexza Pharmaceuticals, Mountain View, CA, USA. Wenxiang Zeng: Employee, Alexza Pharmaceuticals, Mountain View, CA, USA. Emilie van Brummelen: Employee, CHDR, Leiden, the Netherlands. Tatjana Naranda: President and COO, Alexza Pharmaceuticals, Mountain View, CA, USA. Geert Jan Groeneveld: CSO/CMO at CHDR and professorship at LUMC, Leiden, the Netherlands.

## Supporting information


**Figure S1** Overview of study designs.

**Figure S2** CONSORT flow diagram for study part A.

**Figure S3** CONSORT flow diagram for study part B.

**Figure S4** CONSORT flow diagram for study part C.

**Table S1** PK parameters of apomorphine after single‐dose administrations of 1 mg AZ‐009 inhalation and 2 mg subcutaneous (sc) injection to healthy volunteers.

**Table S2** PK parameters of apomorphine after single‐dose administrations of 2 and 3 mg AZ‐009 to healthy volunteers (part B) and 2, 3, and 4 mg AZ‐009 to Parkinson's disease patients (part C).

## Data Availability

The data that support the findings of this study are available from Alexza Pharmaceuticals Inc.. Restrictions apply to the availability of these data, which were used under license for this study. Data are available from the authors with the permission of Alexza Pharmaceuticals Inc.
