# Supplementary material for: A Randomized Trial Assessing the Safety, Pharmacokinetics, and Efficacy During Morning Off of AZ‐009
Source: Mov Disord. 2022 Jan 20;37(4):790–8. doi: 10.1002/mds.28926 (PMC9306836; doi:10.1002/mds.28926)
Supplement: Supplementary file 3 — Figure S3 CONSORT flow diagram for study part B. [file MDS-37-790-s004.docx]

**Supplemental figure 3.** CONSORT flow diagram for study part B.
