# Supplementary material for: A Randomized Trial Assessing the Safety, Pharmacokinetics, and Efficacy During Morning Off of AZ‐009
Source: Mov Disord. 2022 Jan 20;37(4):790–8. doi: 10.1002/mds.28926 (PMC9306836; doi:10.1002/mds.28926)
Supplement: Supplementary file 5 — Table S1 PK parameters of apomorphine after single‐dose administrations of 1 mg AZ‐009 inhalation and 2 mg subcutaneous (sc) injection to healthy volunteers. [file MDS-37-790-s005.docx]

**Supplemental table 1.** PK parameters of apomorphine after single-dose administrations of 1 mg AZ-009 inhalation and 2 mg subcutaneous (sc) injection to healthy volunteers.

|  | **2 mg sc apomorphine**  (N=8) | **1 mg AZ-009**  (N=8) |
| --- | --- | --- |
| **T_max_ (min)** | | |
| Median (range) | 30 (20 – 60) | 1 (1 - 2) |
| **C_max_ (ng**·**mL^-1^)** | | |
| Mean (SD) | 8.6 (3.1) | 14.3 (7.7) |
| Median (range) | 6.8 (5.2 – 12.9) | 14.5 (3.7 – 23.7) |
| **AUC_0-inf_ (****h**·**ng**·**mL^-1^)** | | |
| Mean (SD) | 11.4 (2.6) | 4.9 (2.3) |
| Median (range) | 11.8 (7.6 – 14.6) | 4.9 (2.3 – 9.1) |
| **T_1/2_ (min)** | | |
| Mean (SD) | 55 (22) | 39 (7) |
| Median (range) | 49 (33 – 95) | 39 (25 - 48) |

PK, pharmacokinetics; T_max_, time to maximum plasma concentration; C_max_, maximum plasma concentration; SD, standard deviation; AUC_0-inf_, area under the plasma concentration-time curve from zero to infinity; T_1/2_, apparent terminal elimination half-life.
