# Supplementary material for: A Randomized Trial Assessing the Safety, Pharmacokinetics, and Efficacy During Morning Off of AZ‐009
Source: Mov Disord. 2022 Jan 20;37(4):790–8. doi: 10.1002/mds.28926 (PMC9306836; doi:10.1002/mds.28926)
Supplement: Supplementary file 6 — Table S2 PK parameters of apomorphine after single‐dose administrations of 2 and 3 mg AZ‐009 to healthy volunteers (part B) and 2, 3, and 4 mg AZ‐009 to Parkinson's disease patients (part C). [file MDS-37-790-s002.docx]

**Supplemental table 2.** PK parameters of apomorphine after single-dose administrations of 2 and 3 mg AZ-009 to healthy volunteers (part B) and 2, 3, and 4 mg AZ-009 to Parkinson’s disease patients (part C).

|  | **Healthy volunteers (Part B)** | | **Parkinson’s disease patients (Part C)** | | |
| --- | --- | --- | --- | --- | --- |
|  | **2 mg AZ-009**  (N=6) | **3 mg AZ-009** (N=6) | **2 mg AZ-009**  (N=7) | **3 mg AZ-009** (N=6) | **4 mg AZ-009** (N=6) |
| **T_max_ (min)** | | |  |  |  |
| Median (range) | 1 (1 – 10) | 1 (1 – 5) | 2 (2 – 6) | 2 (2 – 2) | 3 (2 – 5) |
| **C_max_ (ng**·**mL^-1^)** | | |  |  |  |
| Mean (SD) | 16.2 (11.1) | 25.0 (9.5) | 12.0 (6.8) | 25.3 (11.0) | 26.5 (16.6) |
| Median (range) | 15.2 (1.3 – 29.3) | 26.6 (11.0 – 38.2) | 10.4 (4.4 – 22.7) | 29.4 (6.0 – 36.7) | 23.6 (10.3 – 54.2) |
| **AUC_0-inf_ (h**·**ng**·**mL^-1^)** | | |  |  |  |
| Mean (SD) | 5.3 (3.1) | 11.8 (5.4) | 5.1 (1.5) | 12.6 (4.5) | 11.3 (5.1) |
| Median (range) | 5.3 (0.7 – 9.9) | 12.7 (2.5 – 17.6) | 5.0 (3.3 – 7.1) | 14.3 (3.7 – 15.6) | 10.3 (6.5 – 20.6) |
| **T_1/2_ (min)** | | |  |  |  |
| Mean (SD) | 38 (4) | 40 (15) | 38 (10) | 42 (3) | 40 (5) |
| Median (range) | 39 (32 – 42) | 35 (28 – 68) | 38 (20 – 50) | 42 (38 – 45) | 39 (34 – 48) |

PK, pharmacokinetics; T_max_, time to maximum plasma concentration; C_max_, maximum plasma concentration; SD, standard deviation; AUC_0-inf_, area under the plasma concentration-time curve from zero to infinity; T_1/2_, apparent terminal elimination half-life.
